# Furnace

**Published:** 1848-04

**Authors:** A. B. Hoy


*Fayetteville, Ark., Dec.* 18*th*, 1847.

Prof. James Taylor,

*Dear Sir:* You ask me to send you a description of my furnace, for publication in the Register. I cheerfully send it, yet at the same time feel that there is much improvement to be made in it, but as it is, I have derived much benefit from it, and if the profession at large do likewise, then is my object accomplished. Give it a fair test, then decide upon its merits. The advantages of it will be much greater to those of the profession, who, like myself, live in the back woods, where patent blow-pipes, and self operating alcohol lamps, &c., are not to be had; where, with a furnace manufactured out of a joint of a stove pipe, you will be enabled to solder a full set of teeth, with but little use of blow-pipe and lamp, but enough.

A sheet iron cylinder, 8 inches diameter, 18 inches long, to stand at an inclination of about 70 degrees, a sheet iron bottom, screwed on to a piece of plank, 12 inches diameter; the top, (on the lower side,) scolloped out, to the depth of four to five inches, arranged with a piece to slide, up or down, so as to make the cylinder whole when you make the fire, and put in your job, to heat up, or when you wish to take it out for soldering, slide down, that you may be able to take hold with your *tongs*, in the proper position. 9 or 10 inches from the top, punch holes, and make a network of wire half an inch in the mesh. Two and a half inches from the bottom, on the upper side, cut a small air passage, three inches by two, to which fix a slide door, moveable at pleasure, which will regulate the draft as you wish. Upon these slides you may fix a small knob for convenience. Now you have the furnace, next take a band of sheet iron $1\frac{3}{4}$ inch wide, long enough to make it large enough to receive a job when it is made into a ring and riveted, then dip in about half the depth all around, and bend down the points making a bottom. Now you have a cup for the reception of the plaster and sand, into which you place your job covering it over handsomely, and after drying it *thoroughly* putting on the solder, &c. Then you are ready for work, your tongs should be made with a very wide bole spring, easy, points 2 inches long, turned at right angles. Now take some coals of fire, and kindling stuff, place in the furnace, upon which put charcoal, then your job then more coal. Watch the heating, and when in your judgment it has enough (which can only be learned by experience) pick it up with your tongs, having your lamp in readiness, touch the flame to the backs of solder, and in a moment all is done. Then empty out the fire and lay in the job to cool off.

If by accident the solder should drop off of any part, you can replace it and heat up again without any injury to the teeth. For small pieces you may have smaller furnaces, which would be more convenient.

I am, with very great respect,

Your humble servant and friend,

A. B. HOY.

